# Rye kernel breakfast increases satiety in the afternoon - an effect of food structure

**DOI:** 10.1186/1475-2891-10-31

**Published:** 2011-04-11

**Authors:** Hanna Isaksson, Allah Rakha, Roger Andersson, Helena Fredriksson, Johan Olsson, Per Åman

**Affiliations:** 1 Department of Food Science, Swedish University of Agricultural Sciences, P.O. Box 7051, SE-750 07 Uppsala, Sweden; 2 Lantmännen R&D, P.O. Box 30192, SE-104 25 Stockholm, Sweden; 3 KPL Good Food Practice AB, Dag Hammarskjölds väg 10B, SE-751 83 Uppsala, Sweden

## Abstract

**Background:**

The structure of whole grain cereals is maintained to varying degrees during processing and preparation of foods. Food structure can influence metabolism, including perceived hunger and satiety. A diet that enhances satiety per calorie may help to prevent excessive calorie intake. The objective of this work was to compare subjective appetite ratings after consumption of intact and milled rye kernels.

**Methods:**

Two studies were performed using a randomized, cross-over design. Ratings for appetite (hunger, satiety and desire to eat) were registered during an 8-h period after consumption of whole and milled rye kernels prepared as breads (study 1, n = 24) and porridges (study 2, n = 20). Sifted wheat bread was used as reference in both study parts and the products were eaten in iso-caloric portions with standardized additional breakfast foods. Breads and porridges were analyzed to determine whether structure (whole vs. milled kernels) effected dietary fibre content and composition after preparation of the products. Statistical evaluation of the appetite ratings after intake of the different breakfasts was done by paired t-tests for morning and afternoon ratings separately, with subjects as random effect and type of breakfast and time points as fixed effects.

**Results:**

All rye breakfasts resulted in higher satiety ratings in the morning and afternoon compared with the iso-caloric reference breakfast with sifted wheat bread. Rye bread with milled or whole kernels affected appetite equally, so no effect of structure was observed. In contrast, after consumption of the rye kernel breakfast, satiety was increased and hunger suppressed in the afternoon compared with the milled rye kernel porridge breakfast. This effect could be related to structural differences alone, because the products were equal in nutritional content including dietary fibre content and composition.

**Conclusions:**

The study demonstrates that small changes in diet composition such as cereal grain structure have the potential to effect feelings of hunger and satiety.

**Trial registration:**

This trial was registered at clinicaltrials.gov as NCT01042418.

## Background

A high intake of dietary fibre and whole grain foods is associated with a lowered risk of body weight gain, partly owing to an increased feeling of satiety preventing excess energy intake [1,2]. Satiety, or the feeling of fullness, is primarily a result of neural and hormonal signalling throughout the gastrointestinal tract with the purpose of regulating further intake and optimizing digestion and absorption [3]. The influence on perceived satiety varies according to several chemical and physical properties of foods. Food structure changed by milling of cereal grains is one attribute that influences the metabolic response [4]. The botanical structure of cereal grains such as wheat, oats, and rye is maintained to varying degrees during food processing. The grains can be cracked, cut, rolled or milled before being used in a wide range of whole grain products including breads, pasta and breakfast cereals. Rye is a cereal traditionally consumed as whole grain in the Eastern and Nordic Countries. Whole grain rye contains about 20 g dietary fibre per 100 g, which is higher than in other commonly consumed grains [5].

A few studies have investigated the impact of cereal grain structure on satiety. Two studies on whole grain wheat showed that breads including whole kernels increased satiety more than breads made with whole grain flour, at 15 min after intake [6] and during 90 min after intake [7]. When whole kernels, cracked kernels, coarse and fine wheat flour were prepared as oven-baked products, no significant difference on satiety was seen over a time period of 2 h [8]. Also, no variation on postprandial satiety measured for 3 h was found in a comparison of whole and milled barley kernels prepared as porridges [9]. Both studies does however show that grain structure influence the metabolic response, in that glucose and insulin levels were lower after intake of kernels than after flour. Satiety is commonly measured in a period of 0.5-3 hours and longer-term effects are rarely explored. An overnight effect was studied by serving breads made with cut or whole barley kernels as evening meals [10]. The satiating effect of the products did not differ when measured in the following morning. We have previously shown an increased satiety lasting for up to 8 hours after intake of rye porridge made from whole grain rye flakes, compared to sifted wheat bread [11]. In an additional study [12], rye bread made from finely ground flour, resulted in an increased satiety during the first 4 h after consumption. However, in this study there was no difference in satiety in the afternoon. Together, this suggests a potential of cereal structure (flakes vs. flour) to influence satiety up to 8 hours after intake.

The aim of the present study was to investigate if variation in rye grain structure influences perceived appetite during 8 hours. Whole rye kernels and milled rye kernels were compared in a two-part study. The first part compared breads made with milled and whole rye kernels, and the second part porridges of milled and whole rye kernels. Both study parts included a reference breakfast with sifted wheat bread. Special efforts were taken to analyze potential differences in dietary fibre content and composition between the products made from whole or milled rye kernels.

## Methods

### Subjects

Subjects were recruited by sending an inquiry via e-mail to students and employees at the Swedish University of Agricultural Sciences, Uppsala, Sweden. Volunteers were further informed in person about the study procedures and screened to determine their eligibility for inclusion. The criteria for inclusion were the following: man or woman aged between 20 and 60 years; body mass index (BMI) 18-27 kg/m^2^; habit of consuming breakfast, lunch and dinner every day; and willingness to comply with the study procedures. Exclusion criteria were the following: intake of medicine likely to affect appetite or food intake; any medical condition involving the gastrointestinal tract; eating disorder; smoking; consumption of more than three cups of coffee per day; change in body weight more than 10% during three months prior to screening; consumption of any restricted diet such as vegan, gluten-free, slimming, etc.; pregnancy, lactation or wish to become pregnant during the study period.

Weight and height were measured with the subjects wearing light indoor clothing but no shoes and the data obtained were used to calculate BMI. Capillary blood samples were taken in the fasting state for analysis of glucose, thyroid-stimulating hormone, and haemoglobin and alanine aminotransferase.

Initial recruitment started in October 2008 for the bread study, which was performed over 5 weeks starting in early November 2008. All subjects participating in the bread study were invited to the porridge study, which was performed over 5 weeks starting in early April 2009. Written informed consent was obtained from each subject. The study was carried out in compliance with the Helsinki Declaration and was approved by the Ethics Committee at Uppsala University, Sweden.

### Study design

A randomized, cross-over design was used, with three treatments in each of the two study parts. The test sessions (08:00-16:00) were separated by between 5 and 15 days during which the subjects were asked to follow their ordinary diet. The subjects were not informed about the aim of the study or about the content of the test products. On the day prior to each test day, subjects were instructed not to conduct any vigorous physical activity or drink any alcoholic beverages. They were also instructed to eat dinner before 20:00 and to keep the dinner meal similar before each test occasion. They were instructed not to eat or drink anything but water after 20:00.

Upon arrival in the morning (08:00) on test days, each subject was served one of the test breakfasts. During eating, they were seated in a dining area and allowed to make conversation but not regarding anything relating to the study. The subjects were seated at separate tables according to which breakfast they were served, so that those sitting together were eating the same type of breakfast. At the beginning of each session, subjects were asked to fill out questionnaires regarding food and beverage consumption and exercise pattern from the previous evening. After finishing the breakfast meal, the subjects received a lunch bag to be eaten at 12:00 and were free to return to their normal daily activities, such as office work or studies. During the day, they were not allowed to conduct any heavy physical activity, including brisk walking, and restricted to stay within the campus area. They were not allowed to eat or drink anything except foods included in the study diet and moderate amounts of water. A compliance form was filled out by each subject during the day to ensure that instructions were followed.

Subjective feelings of appetite were assessed every 30 min, starting just before breakfast at 08:00 and continuing until 16:00. The data were collected using a specially designed program [13] on a handheld computer, model Palm z22 (Palm Inc, Sunnyvale, USA). At each appetite recording, an alarm went off to remind the subject. The following three questions were presented in sequence: 'How hungry do you feel right now?', 'How full do you feel right now?' and 'How strong is your desire to eat right now?', along with three respective scales marked at opposite ends: Not at all hungry/Extremely hungry, Not at all full/Extremely full, Not at all strong/Extremely strong. The computer mimics the use of pen and paper as it is operated by tapping the screen with a rubber pen. When entering the score, the subjects were prevented from referring to a previous rating. Like the conventional 100 mm visual analogue scale [14], the computerized system translates the mark that the subject makes along the scale to a number between 0 and 100.

At 16:00, the subjects returned to the dining area to hand in the palm computers and compliance forms. They were asked about any complications during the day. In the porridge study, the subjects wore pedometers to count steps taken between 08:00 and 16:00.

### Rye and wheat material

For the bread study, rye kernels commercially used in breads were used (Lantmännen Cerealia, Vejle, Denmark). These untreated kernels were slightly cracked by rolling (2 mm) in order to decrease the time for water absorption and soften the consistency. Rye kernels developed for use as a rice replacer (Lantmännen Cerealia, Järna, Sweden) were used in the porridge study. These kernels were steamed before being lightly cracked by rolling (1.8 mm) in order to decrease cooking time. Both types of rye kernels described above were used whole and milled to fine whole grain flours using a laboratory mill (model 3100, screen size 0.8 mm, Perten Instruments, Stockholm, Sweden). Commercial sifted wheat flour of high quality (Bagerivetemjöl, Nordmills, Malmö, Sweden) was used in all breads, including the wheat reference bread.

### Breads

Three types of bread were made in a small-scale bakery; bread with whole rye kernels, bread with milled rye kernels and wheat reference bread. To create breads with acceptable palatability and soft texture, rye comprised 50% of the total amount of flour. A higher content of rye resulted in compact, crumbly breads, with low ability to prove during fermentation.

Soaked rye kernels were prepared by adding rye kernels (900 g) to a pot of boiling water (1350 g) and left to soak under lid for 60 min. Soaked milled rye kernels were prepared by adding boiling water (1350 g) to milled rye kernels (900 g) in a plastic container. After mixing by hand, the blend was covered with plastic wrapping and left to soak for 60 min.

Both of the rye breads were then baked according to the same procedure. The soaked rye was mixed with sifted wheat flour (900 g), fresh baker's yeast (75 g), gluten (150 g), rape seed oil (90 g), syrup (90 g) and salt (27 g) and kneaded for 7 min using a mixer (Varimixer, Bjørn, Wodschow & Co, Brøndby, Denmark). The dough was left to rest at room temperature (~22°C) for 10 min, then weighed and divided equally into six loaves. The loaves were left to prove for 30 min at 34°C and then baked at 200°C for 40 min.

The wheat reference bread was made by mixing sifted wheat flour (1800 g), water (1050 g, 25°C), fresh baker's yeast (75 g), gluten (150 g), rapeseed oil (90 g), syrup (90 g) and salt (27 g) for 7 min. The dough was left to rest at room temperature for 10 min and then weighed and divided equally into six loaves. The loaves were left to prove at 34°C for 30 min and then baked at 200°C for 30 min. The wheat bread used as reference in the porridge study was baked in the same way, except that gluten was excluded.

After cooling for approximately 2 h, the ends of the loaves were removed. Test portions were weighed, packed individually into plastic bags and stored frozen until the night before each test breakfast.

### Porridges

The porridge of whole rye kernels was prepared by adding rye kernels (66 g) and salt (0.75 g) to boiling water (155 g) and simmering gently for 11 min. By then all water was absorbed by the kernels.

The porridge of milled rye kernels was made by adding milled rye (66 g) kernels to boiling water (350 g), removing the pot from the heat and whipping the contents for 4 min to a smooth porridge.

### Meals

Food intake was standardized in terms of type, amount and timing during the 8-h test period. The test breads were served with the following additional foods: 10 g margarine (40% fat), 24 g cheese (28% fat), 200 g orange juice and one cup of tea or coffee. In the porridge study, the following additional breakfast foods were used: 10 g margarine (40% fat), 60 g lingon berry jam and 200 g milk (1.5% fat). The subjects were allowed one cup of tea or coffee with up to two sachets of milk (20 g, 1.5% fat). Choice of hot drink was individually standardized between test occasions. No sugar or sweetener was allowed. The breakfast meals provided 2550 kJ in the bread study and 1850 kJ in the porridge study. The standardized lunch used in both study parts consisted of a ready-made vegetarian pasta dish of 400 g providing 2040 kJ, 21 g of protein, 64 g of carbohydrates and 16 g of fat per dish (Pasta pomodoro e mozarella, Gooh!, Stockholm, Sweden) and was served with 40 g of cherry tomatoes and 40 g of cucumber. The breakfast and lunch meals had to be consumed entirely and within 30 min. At 14:00, the subjects ate an apple and could choose to drink a cup of tea or coffee, the drink was then kept identical on the following test days.

### Chemical analysis

Breads and porridges were freeze-dried, homogenized in a cyclone sample mill (Retsch, Haan, Germany) and stored at -20°C. In order to obtain representative samples of the porridges, samples were collected every test week. All the samples were analyzed at least in duplicate and results are reported on a dry matter basis. Dry matter was determined by drying the samples at 105°C for 16 h according to the AACC method 44-15A [15]. Extractable and unextractable dietary fibre was analyzed according to the Uppsala method [16]. The β-glucan content was determined using the Megazyme (Bray, Ireland) K-BGLU kit [17]. Fructan was quantified by a spectrophotometric method using the K-FRUC kit (Megazyme, Bray, Ireland) [18]. As the samples might contain raffinose-type oligosaccharides, they were treated with α-galactosidase. Arabinoxylan weight-average (*M_w_*) and number-average (*M_n_*) molecular weights were determined using high performance size exclusion chromatography coupled with multiple angle laser light scattering and refractive index detector [5]. Arabinoxylan fractions with retention time 30-44 min were included in the results, as their molecular weight could be calculated with precision. Calcofluor average molecular weight of β-glucan (*M_cf_*) was analyzed using size exclusion chromatography with fluorescence detection and Calcofluor concentration 0.0025% [19]. Molecules with molecular weight less than 10^4 ^g/mol are not detected and were thus excluded from the results. Fructan molecular weight distributions were determined by high performance anion exchange chromatography coupled with amperometric detection [20]. The samples were extracted with 80% ethanol to solublize fructan and inactivate enzymes. For all components analyzed, the deviation between two analytical repeats was less than 5%.

### Statistical analysis

Ratings for satiety, hunger, and desire to eat were analyzed using Minitab (version 15, LEAD technologies, inc, USA). The level of significance was set at p < 0.05. Missing values (< 1%) were replaced by an average of the ratings for the same point in time and treatment. ANOVA was performed as paired t-tests and Tukey comparisons using subjects as random effect and type of breakfast and time points as fixed effects. Separate analyses were performed for morning (08:30-12:00) and afternoon ratings (12:30-16:00).

## Results

### Subjects

Of the 27 subjects recruited initially, 24 complied with the study procedures and completed the bread study. The three drop-outs were caused by illness (not related to the study), energy requirements exceeding those given in the standardized study diet and not meeting the health criteria. In the porridge study, 20 subjects of the 23 recruited initially met the criteria and completed the study. The three drop-outs were caused by failure to comply with the study procedures. All subjects were within a healthy range of measured health parameters (Table 1). Results from pedometer readings showed that the mean number of steps taken between 08:00 and 16:00 was not significantly different between the treatments.

**Table 1:** Subject characteristics

	**Bread study^b^**			**Porridge study^c^**		
		
**Measurement^a^**	**Mean**	**± SD**	**Range**	**Mean**	**± SD**	**Range**
	
Age (y)	25	8	20-55	24	8	21-56
BMI (kg/m^2^)	22.7	2.6	19.3-28.9	22.2	2.1	19.3-26.8
Fasting glucose (mmol/L)	4.9	0.3	4.3-5.6	4.7	0.4	3.9-5.6
Thyroid-stimulating hormone (mlE/L)	1.5	0.5	0.9-2.6	1.6	0.7	0.9-3.9
Haemoglobin (g/L)	133	8.2	121-152	133	6.5	121-142
Alanine aminotransferase (μkat/L)	0.4	0.2	0.2-1.1	0.4	0.1	0.2-0.7

^a ^Normal range for healthy individuals: fasting glucose 4.0-6.1 mmol/L; thyroid-stimulating hormone 0.3-4.0 mlE/L; hemoglobin in men 130-170, in women 120-150 g/L; alanine aminotransferase in men 0.15-1.10, in women 0.15-0.75 μkat/L.

^b ^n = 24 (22 F, 2 M)

^c ^n = 20 (20 F)

### Test products

Within each study part, test products were served in portions providing similar caloric, protein, fat and available carbohydrate content (Table 2), together with standardized additional foods. The rye products contained more dietary fibre and portion size was higher mainly due to higher water content.

**Table 2:** Weight, energy and nutrient content per portion of the test products as served

**Variables**	**Wheat reference bread**	**Bread with milled rye kernels**	**Bread with whole rye kernels**	**Wheat reference bread**	**Porridge of milled rye kernels**	**Porridge of whole rye kernels**
	
Weight (g)	144	158	156	86	350	137
Energy (kJ)^a^	1650	1650	1600	950	950	900
Water (g)^b^	48	58	61	32	295	85
Protein (g)^b^	15.8	15.8	15.6	6.9	5.7	5.6
Fat (g)^b^	5.8	5.7	6.2	3.7	1.5	1.5
Available carbohydrate (g)^c^	67	63	60	38	40	38
Total dietary fibre^d ^(g)	5.3	13.0	11.0	3.4	11.7	10.7

^a^Calculated according to the standard food energy conversion factors: protein 17 kJ/g, available carbohydrate 17 kJ/g and fat 37 kJ/g. The standard energy value of 8.4 kJ/g was used to estimate contributing energy from dietary fibre.

^b^Analyzed in duplicate by Eurofins, Lidköping, Sweden

^c^Calculated by difference (total weight minus water, protein, fat, ash and total dietary fibre).

^d^Calculated as the sum of fructan and dietary fibre analyzed by the Uppsala method at the Swedish University of Agricultural Sciences, Uppsala, Sweden

The breakfast and lunch meals were well liked and finished completely within 30 min. No adverse events were recorded. Anecdotally, many commented on the large portion size of the porridges, especially the milled kernel porridge.

The total dietary fibre content including fructan in the breads with whole rye kernels (12%) and milled rye kernels (13%) differed only slightly (Table 3). Likewise, total dietary fibre content in the two porridges was similar: 21% and 22% in the whole and milled kernel porridge, respectively. The difference was probably a result of formation of resistant starch (type III, retrograded amylose). Amounts of the other dietary fibre components (arabinoxylan, arabinogalactan, β-glucan, fructan and klason lignin) were similar for both rye breads and porridges, respectively.

**Table 3:** Dietary fibre content and composition in breads and porridges made from whole and milled rye kernels, and two reference breads (% of dry matter)

**Dietary fibre component**	**Wheat reference bread**	**Bread with milled rye kernels**	**Bread with whole rye kernels**	**Wheat reference bread**	**Porridge of milled rye kernels**	**Porridge of whole rye kernels**
	
Dietary fibre						
total^a^	5.5	13.0	11.6	6.1	21.6	20.7
extractable^b^	1.8	4.1	4.1	1.8	7.8	7.7
unextractable	3.7	8.9	7.5	4.4	13.8	13.1
Arabinoxylan^c^						
total	1.6	4.4	4.4	1.9	8.8	8.5
extractable	0.9	1.8	1.7	0.9	2.7	2.6
unextractable	0.7	2.6	2.7	1.0	6.1	6.0
Arabino-galactan^c^	0.2	0.2	0.2	0.2	0.1	0.2
β-glucan	0.3	1.0	1.0	0.4	2.0	1.9
Cellulose and resistant starch^d^	1.4	3.7	2.6	1.6	3.5	2.8
Fructan	0.5	1.6	1.7	0.5	4.4	4.3
Klason lignin	0.8	1.1	0.9	0.8	1.5	1.6

^a^Total dietary fibre calculated as the sum of fructan and total dietary fibre analyzed by the Uppsala method.

^b^Extractable dietary fibre calculated as the sum of fructan and extractable dietary fibre analyzed by the Uppsala method.

^c^Calculated from arabinose, xylose and extractable galactose residue values assuming that the arabinose to galactose ratio is 0.69 in extractable arabinogalactan.

^d^Cellulose and resistant starch calculated as the difference between total β-glucan and the glucose residues analyzed by the Uppsala method.

**Figure 1:**
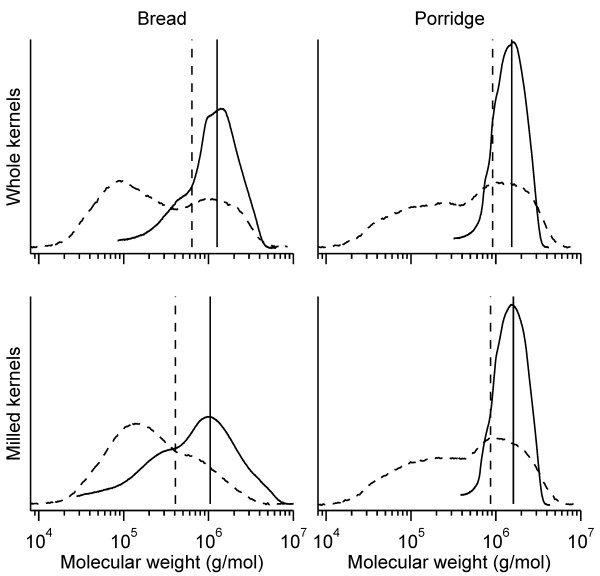
**Molecular weight distributions of extractable arabinoxylan (solid line) and β-glucan (dashed line) in the rye products**. Vertical lines denote weight-average molecular weights.

Slight differences due to milling were observed, as the bread with whole kernels had slightly higher M_w _and M_n _and lower polydispersity index for extractable arabinoxylan than the bread with milled kernels (Table 4). The porridges were similar in molecular weight parameters of extractable arabinoxylan. The molecular weight distribution pattern showed a higher proportion of low molecular mass fraction of extractable arabinoxylan in the bread made from milled rye kernels (Figure 1). Extractable arabinoxylan distribution pattern in the porridges was similar and fell within a narrow range.

**Table 4:** Weight (M_w_) and number (M_n_) average molecular weight and polydispersity index (M_w_/M_n_) of extractable arabinoxylan, and yield and Calcofluor average molecular weight (M_cf_) of extractable β-glucan

**Analysis**	**Wheat reference bread**	**Bread with milled rye kernels**	**Bread with whole rye kernels**	**Wheat reference bread**	**Porridge of milled rye kernels**	**Porridge of whole rye kernels**
	
Arabinoxylan						
*M*_*w *_(10^5^g/mol)	6.15	10.9	13.1	6.13	16.0	15.4
*M*_*n *_(10^5^g/mol)	3.13	3.07	7.38	2.82	13.6	12.8
*M_w_/M_n_*	1.97	3.55	1.78	2.17	1.18	1.20
β-glucan						
Extractability (%)	37	35	25	26	14	14
*M_cf _*(10^5^g/mol)	1.93	3.90	6.18	2.16	8.40	8.88

Extractability of β-glucan was higher in the bread made from milled rye kernels (35%) than in the bread made from whole rye kernels (25%, Table 4), whereas β-glucan extractability was lower in both porridges (14%). The distribution profile showed an elevated fraction of low molecular weight β-glucan in bread made from milled rye kernels (Figure 1). Average molecular weight of extractable β-glucan in bread with whole kernels was higher than in bread with milled rye kernels, indicating higher degradation of β-glucan during bread-making. Extractable β-glucan molecular weight was similar between the porridges.

**Figure 2:**
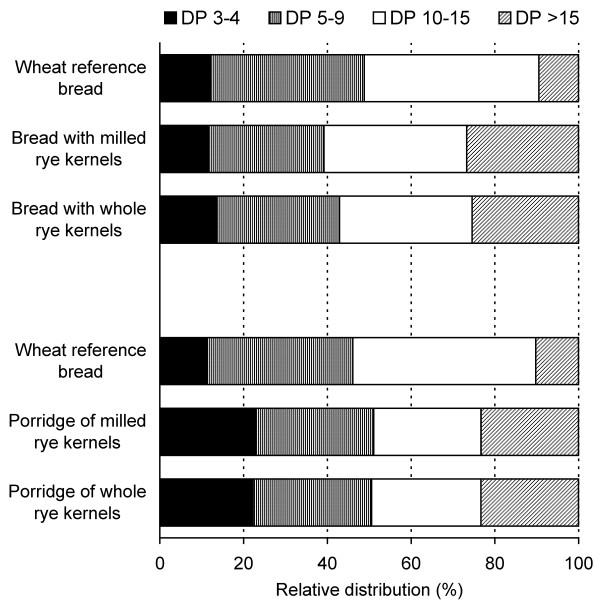
**Relative molecular weight distribution of fructan**. DP = degree of polymerization.

The relative distribution of fructan molecules of varying degrees of polymerization (DP) was very similar in the two porridges (Figure 2). The proportion of high DP fructan was higher in the breads, indicating the disappearance of lower DP fructan.

Taken together, disintegration of the rye kernels had little impact on molecular weight parameters of extractable arabinoxylan, β-glucan and fructan in porridges made from whole kernels and milled rye kernels, while some differences could be seen between the two rye breads.

### Appetite ratings - Bread study

**Figure 3:**
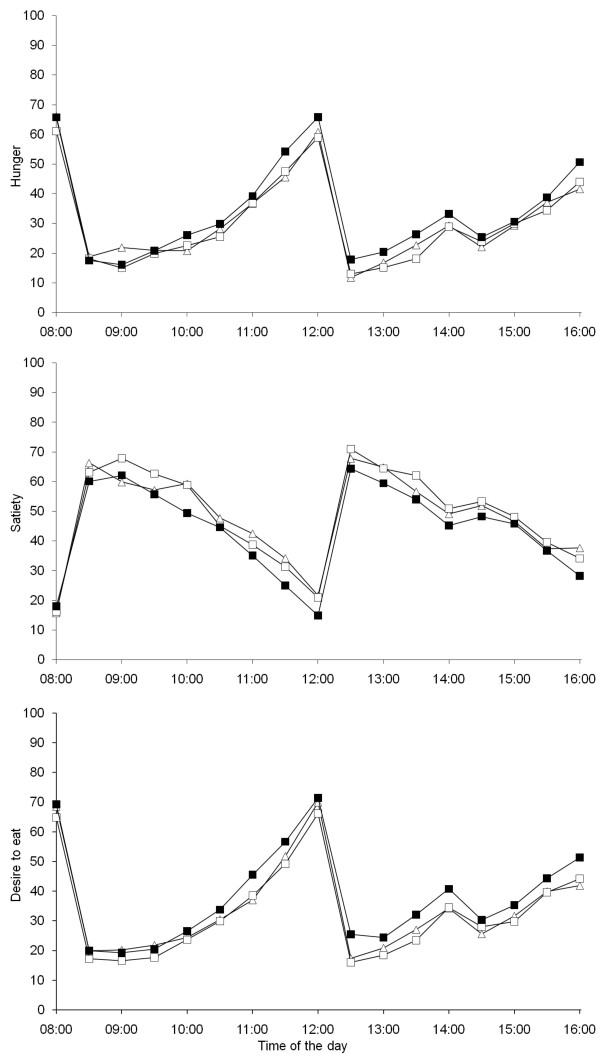
**Appetite ratings (n = 24) during 8 h after consumption of breakfast meals including wheat reference bread (-■-), bread with milled rye kernels (--△--) and bread with whole rye kernels (--□--)**.

Before breakfast, the mean ratings for hunger, satiety and desire to eat were similar between test days (Figure 3). Appetite ratings showed a clear effect of time after breakfast and after lunch, i.e. the ratings visibly demonstrated the subjects' responses of lower hunger and increased satiety directly after a meal and then progressively stronger hunger and weaker feelings of satiety as the time approached the next meal.

**Figure 4:**
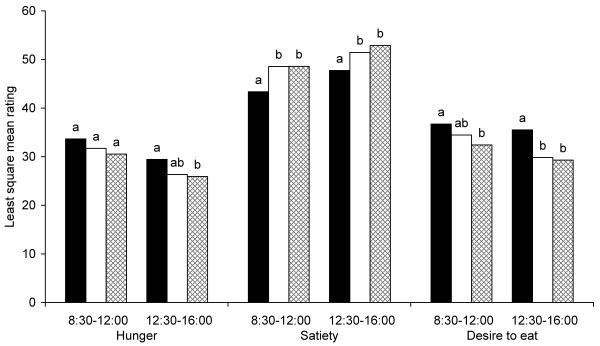
**Appetite ratings (n = 24) for the morning and afternoon time intervals after intake of breakfast meals including wheat reference bread (black); bread with milled rye kernels (white) and bread with whole rye kernels (checked)**. Different letters within time interval and appetite rating indicate significant difference (p < 0.05).

Satiety was rated higher during the morning (08:30-12:00) after intake of both rye bread breakfasts (Figure 3 and 4) compared with the wheat reference bread breakfast. The bread including whole rye kernels also resulted in a reduced desire to eat compared with reference. Hunger ratings did not differ significantly during the morning after intake of the three different breads. During the afternoon (12:30-16:00), however, both rye bread breakfasts resulted in increased satiety and reduced desire to eat compared with the wheat reference bread breakfast. The bread with whole rye kernels also reduced hunger in the afternoon compared with reference. Comparisons between the two rye breads (whole rye kernels vs. milled rye kernels) revealed no significant difference in any appetite measure in either morning or afternoon.

In summary, both of the rye breads resulted in increased satiety compared with the wheat reference, but there was no difference in any of the appetite measures between the two rye breads.

### Appetite ratings - Porridge study

**Figure 5:**
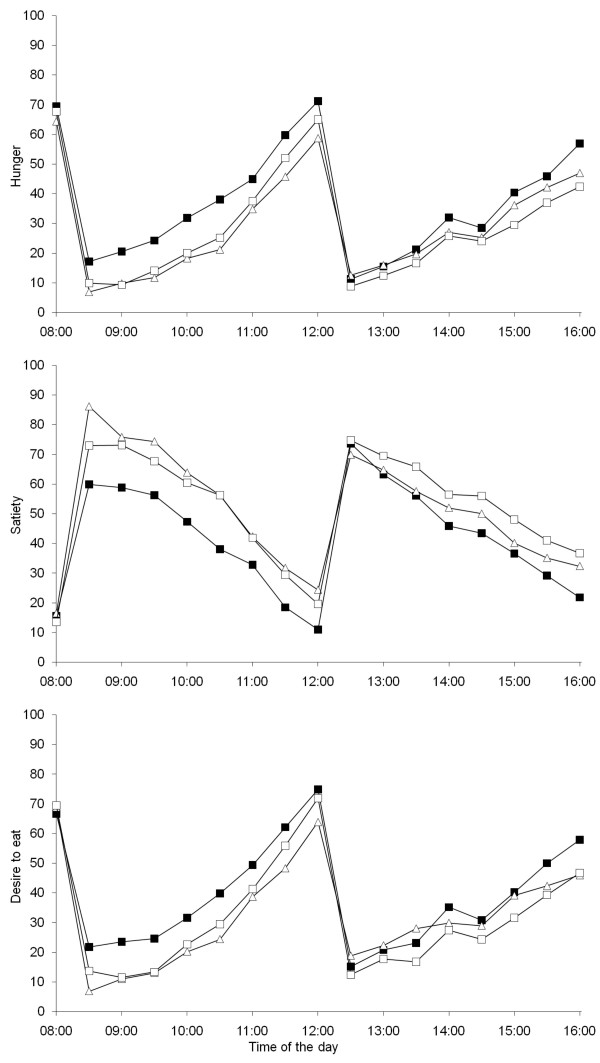
**Appetite ratings (n = 20) during 8 h after consumption of breakfast meals including wheat reference bread (-■-), porridge of milled rye kernels (--△--) and porridge of whole rye kernels (--□--)**.

**Figure 6:**
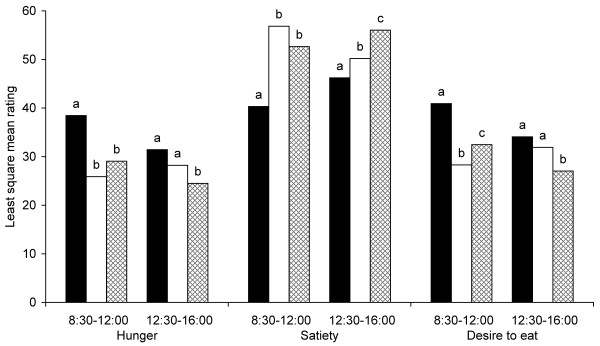
**Appetite ratings (n = 20) for the morning and afternoon time intervals after consumption of breakfast meals including wheat reference bread (black); porridge of milled rye kernels (white) and porridge of whole rye kernels (checked)**. Different letters within time interval and appetite rating indicates significant difference (p < 0.05).

Baseline ratings for hunger, satiety and desire to eat were similar between test days (Figure 5). During the morning (08:30-12:00), both rye porridge breakfasts resulted in increased satiety, reduced hunger and reduced desire to eat compared with the wheat reference bread breakfast (Figures 5 and 6). During the afternoon (12:30-16:00), the effect of breakfasts was still evident; both rye porridge breakfasts resulted in increased satiety compared with the reference breakfast with sifted wheat bread. The porridge made with rye kernels also reduced hunger and desire to eat. Comparisons between appetite ratings after the two rye porridges showed that the one made of whole kernels resulted in stronger satiety, lower hunger and lower desire to eat during the afternoon than that made of milled rye kernels.

## Discussion

The aim of this study was to investigate the effect of rye kernel structure on 8-h subjective appetite. Whole and milled rye kernels were compared in a two-part study. In the first part, the rye material was baked into breads and in the second part used to make porridges. Sifted wheat bread breakfast was used as reference in both study parts.

Both rye bread breakfasts increased postprandial satiety levels compared with the wheat reference bread. This agrees with our previous findings, which showed a decrease in hunger ratings during the 8-h period after consumption of rye bread breakfasts including rye bran and sifted rye flour [12]. Similarly, bread that contained rye bran resulted in higher feelings of satiety during 3 h after intake compared with reference wheat bread [21]. In contrast, no difference was reported in appetite ratings for a small group of obese subjects with metabolic syndrome during 2 h after consumption of whole grain rye bread compared with a combination of wheat and oat bread [22]. These breads were however not compared on an iso-caloric basis but the wheat and oat bread contained slightly more energy.

The current study found no difference between the rye breads with different rye material regarding effects on appetite. Contrasting to previous results showing that bread with wheat kernels (over 80% kernels in total flour) increased satiety more than bread with whole grain wheat flour during 2 h after intake [7].

Test products in appetite rating studies are often not well-characterized. In this study, special efforts were taken to characterize dietary fibre content and structure in the different test products. Processing had only small effects on the dietary fibre content of breads made from whole or milled rye kernels. However, there was slightly higher total dietary fibre content in bread made from milled rye kernels, probably a result of higher resistant starch formation in the crumb during bread-making [23]. These minor differences in dietary fibre composition and molecular weight distribution of extractable dietary fibre between the two rye breads did not result in any differences in appetite, since both breads exerted similar appetite responses.

The fact that both of the rye porridges induced satiety more than the reference bread confirmed results from previous studies [11,21]. Both porridges combine a number of potential satiety enhancing factors. Increased food volume, by inclusion of water, was shown to increase satiety and decrease spontaneous food intake in the short term [24]. In addition, the porridges provided much higher dietary fibre content than the reference bread. Parts of the effect may relate to the viscosity-inducing properties of rye dietary fibre.

In the present study, the weight and volume of the milled kernel porridge was three-fold that of the kernel porridge, resulting in an immediate peak in satiety ratings. An effect that soon disappeared and the two rye porridges had similar effects on satiety during the morning hours. In the afternoon, milling of the rye ingredient seemed to affect appetite, as the whole kernel porridge resulted in a stronger satiety, reduced hunger and reduced desire to eat compared with the milled kernel porridge. Except for the slightly higher resistant starch content in the milled kernel porridge, all other dietary fibre components were similar. Thus, the effects can be solely related to structure.

The mechanism by which the intact kernel porridge induced satiety in the afternoon may be by reducing small intestinal digestion and absorption by forming a physical barrier to digestive enzymes [25], hypothetically providing more material for colonic fermentation resulting in the late increase in satiety. End products caused by colonic fermentation of dietary fibre, such as butyrate, have been suggested to affect satiety. How this effect is mediated is not clear. Suggested mechanisms are stimulated release of satiety hormones (GLP-1, PYY) by L-cells in the colon [26]. An increased fermentation measured by breath hydrogen was recently correlated with increased satiety [27,28]. A higher mean breath hydrogen excretion 4-6 h after consumption of rye kernels compared with oat or wheat kernels indicating early and extensive colonic fermentation [27]. Rye bread intake has also been shown to lower the digestibility of protein, fat and starch in a pig model [29], resulting in increased ileal excretion of macronutrients in ileostomi patients [30].

The present study showed significant differences in appetite ratings for up to 8 h after intake of different breakfast meals. In order to do this, there was a need to standardize energy intake during the whole period of measure. Therefore ad libitum energy intake could not be included in the study design, at least not in the 8-h period after breakfast, which would be most relevant. Appetite ratings have however previously repeatedly been shown to correlate to and predict voluntary energy intake [31-33].

Future studies should explore whether the satiating effect of rye products is persistent over time and if a diet rich in rye products could facilitate weight control.

## Conclusion

This study demonstrates a satiety enhancing effect of whole grain rye foods, breads as well as porridges, compared to an iso-caloric sifted wheat bread. Further, an interesting effect regarding the influence of cereal grain structure was discovered. A breakfast meal including whole rye kernels, resulted in increased feelings of satiety in the afternoon, compared to porridge made of whole grain rye flour. The understanding of the underlying mechanisms to the late response several hours after intake is likely to involve colonic events and requires further investigation. Altogether, the findings show that whole grain rye products improve satiety and lower hunger ratings. This may explain how diets rich in whole grain and dietary fibre protect against overweight.

## Competing interests

The study was supported by the supplier of the material used to make the test products, Lantmännen. HI and HF are employed by Lantmännen R&D, the research and development department of the Lantmännen Group. The authors declare that the data presented in this publication represent a complete, true and faithful representation of the work performed.

## Authors' contributions

HI, RA, HF, JO and PÅ designed the study. HI recruited the subjects and performed the study. AR carried out the dietary fibre characterization. RA and HI analyzed the appetite data. HI and AR wrote the first draft of the manuscript. All authors participated in revising the manuscript, read and approved the final manuscript.
